# The Myb-p300-CREB axis modulates intestine homeostasis, radiosensitivity and tumorigenesis

**DOI:** 10.1038/cddis.2013.119

**Published:** 2013-04-25

**Authors:** S Sampurno, A Bijenhof, D Cheasley, H Xu, S Robine, D Hilton, W S Alexander, L Pereira, T Mantamadiotis, J Malaterre, R G Ramsay

**Affiliations:** 1 Differentiation and Transcription Laboratory, Peter MacCallum Cancer Center,East Melbourne, Victoria, Australia; 2 Sir Peter MacCallum Department of Oncology, Melbourne, Victoria, Australia; 3 Department of Pathology, the University of Melbourne, Parkville, Victoria, Australia; 4 Center National de la Recherche Scientifique, Institut Curie, Paris, France; 5 Walter and Eliza Hall Institute of Medical Research, Parkville, Victoria, Australia; 6 Department of Medical Biology, the University of Melbourne, Parkville, Victoria, Australia

**Keywords:** p300, Myb, CREB, colorectal cancer, Apc^Min/+^

## Abstract

The gastrointestinal (GI) epithelium is constantly renewing, depending upon the intestinal stem cells (ISC) regulated by a spectrum of transcription factors (TFs), including Myb. We noted previously in mice with a *p300* mutation (*plt6*) within the Myb-interaction-domain phenocopied *Myb* hypomorphic mutant mice with regard to thrombopoiesis, and here, changes in GI homeostasis. p300 is a transcriptional coactivator for many TFs, most prominently cyclic-AMP response element-binding protein (CREB), and also Myb. Studies have highlighted the importance of CREB in proliferation and radiosensitivity, but not in the GI. This prompted us to directly investigate the p300–Myb–CREB axis in the GI. Here, the role of CREB has been defined by generating GI-specific inducible *creb* knockout (KO) mice. KO mice show efficient and specific deletion of CREB, with no evident compensation by CREM and ATF1. Despite complete KO, only modest effects on proliferation, radiosensitivity and differentiation in the GI under homeostatic or stress conditions were evident, even though CREB target gene *pcna* (proliferating cell nuclear antigen) was downregulated. *creb* and *p300* mutant lines show increased goblet cells, whereas a reduction in enteroendocrine cells was apparent only in the *p300* line, further resembling the *Myb* hypomorphs. When propagated *in vitro*, *creb*KO ISC were defective in organoid formation, suggesting that the GI stroma compensates for CREB loss *in vivo*, unlike in *Myb*KO studies. Thus, it appears that p300 regulates GI differentiation primarily through Myb, rather than CREB. Finally, active pCREB is elevated in colorectal cancer (CRC) cells and adenomas, and is required for the expression of drug transporter, MRP2, associated with resistance to Oxaliplatin as well as several chromatin cohesion protein that are relevant to CRC therapy. These data raise the prospect that CREB may have a role in GI malignancy as it does in other cancer types, but unlike Myb, is not critical for GI homeostasis.

The epithelium of the small intestine (SI) and colon is the fastest self-renewing tissue in the body. Self-renewal is facilitated by intestinal stem cells (ISCs) near the bottom of the crypts. Gut ISC produce highly proliferating transit-amplifying cells. Maturation of these cells coincides with upward migration and differentiation into either secretory or mucin-secreting goblet cells, hormone peptide-secreting enteroendocrine cells and in the SI a fourth cell type; bactericidal and ISC niche-supporting Paneth cells (PC). When the differentiated cells reach the top of the SI villi or the surface of the colon mucosa, anoikis into the lumen follows.^1^ Most importantly, the gastrointestinal epithelium (GI) is highly sensitive to radio- and chemotherapy,^2^ and is subject to radiation-induced malignancy.^3^

Several pathways involved in the homeostasis of gut have been identified. The best documented is the Wnt pathway definitively shown to be involved in proliferation, the Notch pathway in cell fate and the Ssh/BMP-4 pathway, suggested to contribute to cell survival and -renewal.^1, 4, 5^ The adenomatous polyposis coli (*APC*) gene product serves as the key cancer ‘gatekeeper' that prevents the development of colorectal cancer (CRC) and aberrant activation of the Wnt pathway.^1^ *APC* mutations occur in ∼80% of CRC.^6^ Nevertheless, the precise molecular networks involved in the proliferation and differentiation of GI epithelium under normal, stressed and malignant transformation are far from being defined.

We and others have explored the role of the transcription factor (TF) cyclic-AMP responsive element-binding protein (CREB), finding a correspondence of active phosphor-CREB (pCREB) in proliferating cells in different tissues from diverse organisms. Deletion of the highly evolutionarily conserved *creb* gene alone or in combination with the related family member, *crem*, has shown the need for these TFs in developing and adult brain and in T-cell recovery in irradiated mice.^7, 8, 9^ In addition, *in vitro* and *in vivo* studies show that CREB also has a role in oncogenesis influencing melanoma, T-cell and myeloid leukemia, hepatocellular carcinoma, clear cell sarcoma, lung adenocarcinoma,^10^ as well as CRC.^11^

CREB is a member of the basic region leucine zipper (bZIP) family of TFs. bZIPs bind to the c-AMP responsive element (CRE), a conserved consensus sequence, as homodimers or heterodimers with the closely related family members CREM or ATF1 (Figure 1).^12^ All members contain a kinase-inducible domain (KIX) where the phosphorylation sites are located, a basic domain essential for DNA binding and a leucine zipper dimerization domain.^13^

Multiple external stimuli induce CREB activation via several kinase pathways, including stress pathways.^14^ These signalling pathways lead to phosphorylation of CREB (pCREB) at a conserved serine residue (Ser^133^), triggering interaction with co-activators, including CREB-binding protein (CBP) or p300.^15, 16^ Importantly, p300 and CBP are not only CREB cofactors but also activate additional TFs, including Myb.

An *in vivo* interaction between Myb and p300 was revealed by the identification of the *plt6* mouse strain containing a mutation in the KIX domain.^17^ This mutant mouse phenocopies three distinct hypomorphic *Myb* mutant lines with regard to elevated platelet numbers.^18^ The p300KIX domain interacts with the transactivation domain of Myb. Thus, the interaction between p300 and Myb was shown to be essential for cellular differentiation and lineage hierarchies in hematopoiesis.^17, 19^ Myb is an important TF for proliferation and differentiation of the intestinal epithelium.^20^ The transcriptional coregulator p300 might, therefore, integrate the co-activation and transcriptional activities of both pCREB and Myb, orchestrating finely tuned cellular responses in the GI. In this study, an inducible *creb* knockout (KO) model was used to define the role of CREB in the GI of adult mice in proliferation and differentiation under homeostasis and stress. Extensive pCREB activation was found in *Apc*^*Min/+*^ mouse adenomas. Moreover, in CRC pCREB was detected in most cells associated with elevated CREB-target genes, including CRC-relevant chemotherapy drug Oxaliplatin multidrug transporter, MRP2.

## Results

### *p300*^*plt6/plt6*^ intestines phenocopy *Myb* hypomorphs

As part of a comprehensive ENU mutagenesis screen focused on thrombopoiesis^18^ or hematopoiesis,^19^ Myb emerged as a central factor in these processes.^21^ These observations were consistent with observations made in *Myb*KO mice where definitive hematopoiesis was ablated and embryos died at ed15.^22^ The utility of the *Myb* hypomorphs came to the fore as they allowed the examination of other tissues that also expressed robust levels of Myb, notably the colon and SI in viable adults. Three distinct *Myb* hypomorphs showed defects in the GI, establishing an essential role for Myb in the GI,^20^ and later it was directly required for GI stem cell function.^23^ Two hypomorphs had mutations that impinged upon interaction between Myb and p300/CBP. When the *p300*^*plt6*^ mutation emerged from the ENU screen with a similar phenotype to the *Myb* hypomorphs,^17^ we posed the question whether p300 was also important for homeostasis in the GI.

The expression of p300 and CBP in the GI has been reported, and there are varying views about the relative importance of one over the other in development and homeostasis.^24, 25^ Therefore, we first examined the expression of both p300 and CBP proteins in the GI. (Supplementary Figure 1) Both factors are expressed in the majority of cells in the epithelial layer including post-mitotic cells, highlighting the importance of these transcriptional co-activators in both immature and differentiated cells.

**Figure 1:**
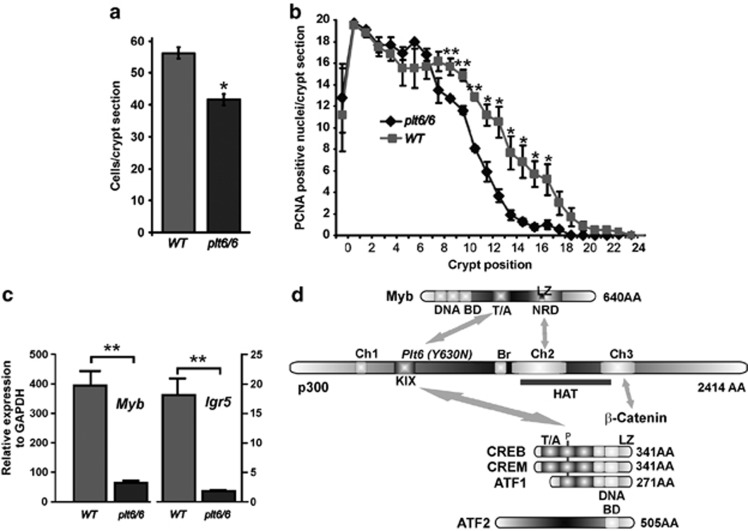
p300 hypomorphic mutant (*plt6/6*) mouse intestinal defects phenocopy *Myb* mutant mice. (**a**) Like *Myb* hypomorphs, *plt6/6* mutant mice have shorter crypts than WT mice (*P*=0.03). (**b**) This reduced crypt length corresponds to reduced proliferation, as determined by PCNA staining at crypts positions from the base (*P*<0.05–0.01). (**c**) Expression of ISC gene *Myb* is significantly reduced in colonic crypts isolated from *plt6/6* mutants compared with *WT* as is Myb target and ISC gene, *lgr5*, *P*<0.01. (**d**) p300 and Myb can be viewed as being part of a larger complex of interacting TFs, including *β*-catenin, and most particularly, closely related CREB family members (CREB, CREM and ATF1) and more distantly related, ATF2. Linear protein lengths are shown in amino acid (AA) residues, and key interaction and function domains (DNA-binding domain (BD), transactivation domain (T/A), negative regulation domain (NRD) with leucine zipper-like domain (LZ), C=conserved homology domains (Ch), kinase interaction domain (KIX) and key conserved phosphorylation site (P). Means±S.E.M. one-tailed *t*-test

Examination of the intestines of *p300*^*plt6/plt6*^ mice showed that the colonic crypts have 30% fewer cells per cross-section than littermate controls (Figure 1a), and there was a premature decline in PCNA+ve (proliferating cells) (Figure 1b). A similar proliferative defect was noted in the SI (data not shown). Of greater interest, however, was that these defects phenocopy those reported by us in *Myb* hypomorphs.^20^ *Myb* RNA expression in purified crypts was significantly reduced in the *p300* hypomorph as was its target gene *lgr5*^23^ (Figure 1c). As p300 interacts with many other TFs, we were mindful that this *p300* mutation might be affecting other KIX-dependent interactions, most notably with the CREB family of TFs. These interactions are depicted in Figure 1d, raising the question of whether CREB, which is expressed in gut epithelium, might be similarly important in the GI for proliferation and crypt homeostasis.

### CREB recombination is specifically and efficiently induced in the GI tract

**Figure 2:**
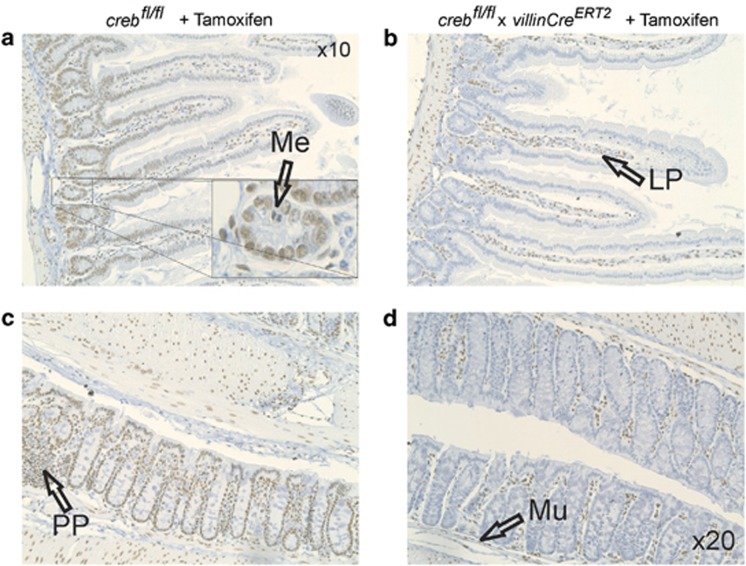
Complete intestinal epithelial deletion of CREB. *creb*^*fl/fl*^ mice (**a** and **c**) and *creb*^*fl/fl*^ x *villinCre*^*ERT2*^ mice (**b** and **d**) were proved chow with Tamoxifen *ad libitum* for 4 weeks before cull, and preparation of colon and SI sections. These were subjected to IHC with an antibody specific to CREB to show complete loss of antigen in epithelial cells associated with crypts, but not lamina propria (LP), Peyer's patches (PP) or muscle (Mu). (Insert panel) Metaphase (Me) figures within the epithelial compartments were CREB−ve (arrow)

In view of studies demonstrating that CREB contributes to proliferation and differentiation of different cell types in multiple non-GI tissue compartments, we deleted CREB in the intestine using a Tamoxifen-inducible CRE-mediated intestine-specific *creb*KO mice line. In these mice, exon 10 was flanked by *loxP* sites, and earlier studies indicated that the deletion of exon 10 results in loss of all CREB polypeptides.^9^ To assess whether CREB deletion occurred in the gut epithelium after 4 weeks of Tamoxifen treatment, immunohistochemistry (IHC) was employed using antibodies against CREB. Control littermates (*creb*^*fl/fl*^+Tamoxifen) showed CREB protein throughout the colonic crypts and the SI extending into the villi (Figures 2a and c). Enterocytes were the predominant CREB+ve cell type, but cells in metaphase were CREB−ve. Importantly, CREB was evident in the lamina propria, lymphoid-rich Peyer's patches and basal stromal regions, particularly where endothelial cells would be expected to reside. The muscularis also contained a few CREB+ve cells. When epithelial cells were examined after Tamoxifen treatment, all crypt and villi exhibited loss of CREB expression (Figures 2b and d). These data indicate that the *creb* deletion (from this point called *creb*KO) was epithelial-specific, pervasive and uniform, but resulted in only modest effects on GI architecture.

### Effect of CREB loss on crypt proliferation and cell fate

**Figure 3:**
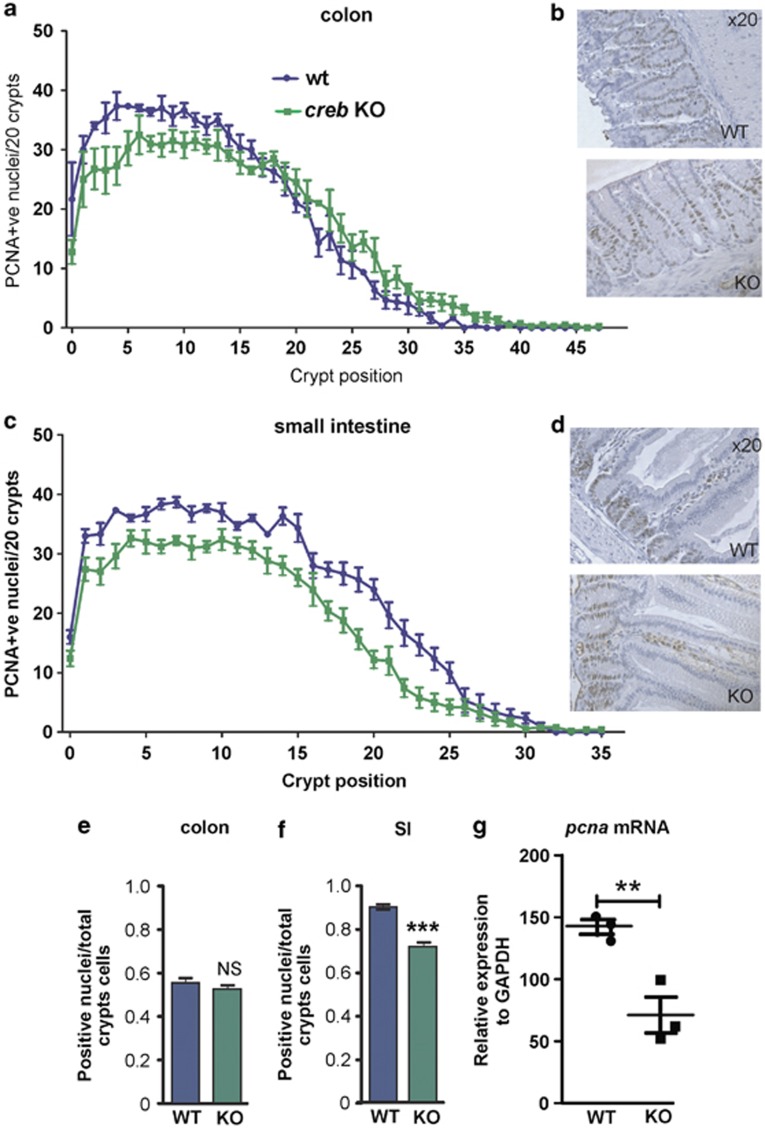
Modestly reduced proliferation in CREB−ve intestinal crypts despite uniform epithelial KO. (**a** and **b**) PCNA IHC was performed and determined at each crypt position from the base, showing a modest reduction in proliferation in the transit-amplifying region of the colonic crypt (**d** and **e**) and throughout the SI crypt. (**e**) Overall, the total PCNA staining in the colon was not significantly different (**f**), whereas a statistically reduced number of PCNA+ve nuclei were found in the SI. (**g**) Relative levels of mRNA for CREB target gene *pcna* was found to be statistically reduced following KO. Means±S.E.M., two-tailed *t*-test. *P=0.01; ***P=0.001

As crypts have a high turnover and *pcna* has been reported to be a CREB target gene,^26^ we examined the proliferative status in WT and *creb*KO intestines by IHC (Figures 3a–d). Overall, there was a reduction in PCNA+ve cells at the crypt base in SI and colon (Figures 3a and c), but this was only significant in the SI when considering the total number of PCNA+ve nuclei (Figures 3e and f) and was confirmed by evaluation of mRNA by qRT-PCR (Figure 3g). To further investigate the *creb*KO colon and SI architecture, we explored the relative numbers of Chromogranin A+ve cells, as CREB regulates the *ChrA* gene^27, 28^ and neuroendocrine cell function.^29, 30, 31, 32^ However, the number of these cells appeared elevated in the SI and significantly increased in the colon in *creb*KO mice. Goblet cells and mucin production, as judged by period acid shift (PAS) histochemistry, was more intense in the *creb*KO colons (Supplementary Figure 2).

### Transcriptionally Active pCREB is not compensated for by other family members

**Figure 4:**
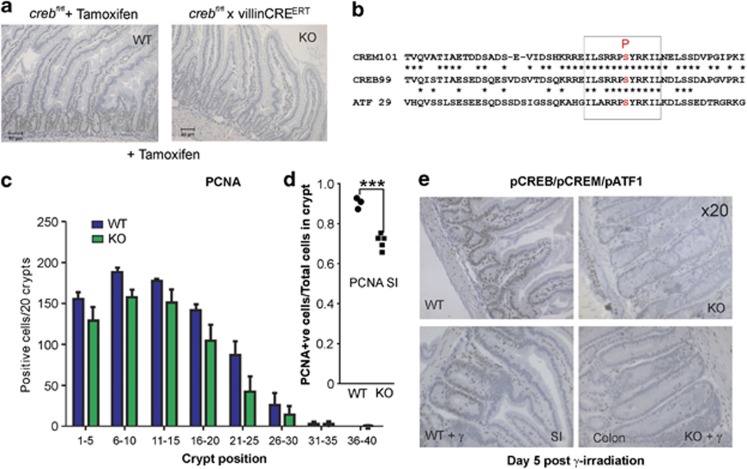
No apparent compensation for CREB loss by family members, CREM and ATF1, including following irradiation. (**a**) WT and KO sections were subjected to IHC with phospho-CREB antibodies to show that under steady state KO, SI epithelial cell showed no signal. (**b**)This antibody also detects closely related phospho-CREM and phospho-ATF1. (**c** and **d**) When mice were exposed to whole-body irradiation and intestines processed, 5 days later it was apparent that the small but significant difference in proliferation observed in unirradiated mice in the SI was not exacerbated with radiation treatment. (**e**) It was notable that the extent of phospho-CREB/CREM/ATF1 was more restricted to the base of crypts in WT mice (SI) and that all three CREB members were not present in the KO crypts (colon) with or without radiation, indicating that these were not induced by radiation damage at this time point

In its active form, CREB is phosphorylated on a key serine residue (ser^**133**^)^14^ (Figure 1d), and pCREB correlates best with proliferation.^33^ Accordingly, GI sections were investigated for the presence of pCREB, where we found a similar distribution of PCNA+ve and pCREB within nuclei. No pCREB staining epithelial cells were evident in the *creb*KO sections (Figure 4a), whereas this active form of CREB, unlike the CREB protein in general, is restricted to the crypts in the SI, consistent with the location of the proliferating transit-amplifying cell and stem cell compartment.^34^ Importantly, due to close structural similarity, the pCREB antibody also recognizes the kinase-inducible domains shared by family members, CREM and ATF1^35^ (Figure 4b), which allows the conclusion to be drawn that the active form of these other members are not co-expressed in the GI epithelium, and therefore, unable to compensate for CREB loss. To extend this analysis further, we also employed an antibody against a more distantly related TF, ATF2 (Figure 1d), finding it to be expressed in a similar manner to pCREB, but in the *creb*KO this antigen is expressed to a weaker extent compared with WT crypts (Supplementary Figure 3).

Defects in proliferation are sometimes revealed or exacerbated by stressing the GI with radiation, as observed with *Myb* mutant mice,^21^ although Myb expression itself was unaffected in the unirradiated CREBKO GI (Supplementary Figure 4). Indeed, CREB has been implicated indirectly influencing radiosensitivity, and its target gene *pcna* is intimately involved in DNA repair.^36^ Mice were thus whole-body irradiated with 13 Gy and allowed to recover for 5 days, at which time point, the mice were killed and GI sections prepared. PCNA IHC showed that in a similar manner to the unirradiated control, there was a significant, but apparently no greater reduction in proliferation in the *creb*KO mice SI (Figure 4c). Even under these stress conditions, pCREB/pCREM/pATF1 were not induced in irradiated GI (Figure 4e), suggesting that PCNA expression under these conditions is in part under the influence of pCREB but that CREB loss does not lead to GI hypersensitivity to radiation, challenging previous views based upon using dominant negative forms of CREB.^36^

### *In vitro* deletion of CREB impedes SI organoid formation

**Figure 5:**
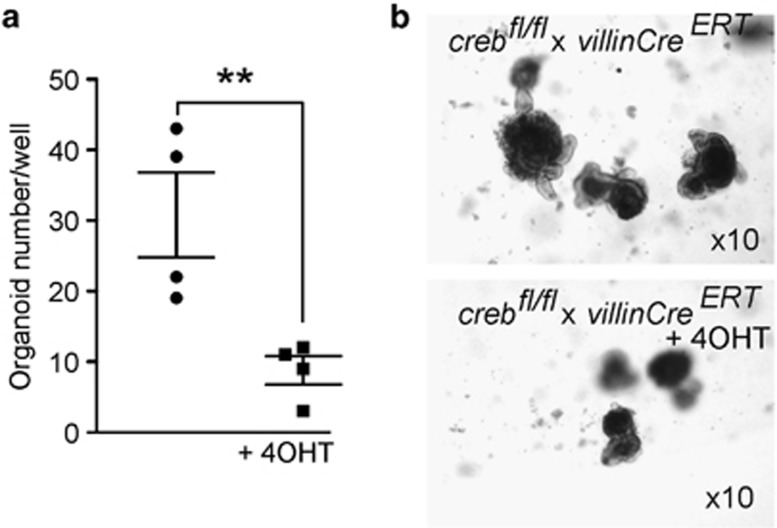
*In vitro* culture of SI organoids reveals a defect in colony-forming ability in KO mice. (**a**) Organoid cultures were initiated from *creb*^*fl/fl*^ x *villinCre*^*ERT2*^ mice and these were exposed to 4OHT to show reduced colony-forming efficiency in the KO cultures; Means±S.E.M. two-tailed *t*-test; **P<0.01. (**b**) Representative images are shown, (mag= × 10)

Having found only modest defects in proliferation of *creb*KO crypts under homeostasis or following challenge with radiation, we decided to quantify the ability of crypt stem/progenitor cells to initiate and form organoids *ex vivo*.^37^ Crypt nests were isolated from *creb/villinCre*^*ERT2*^ mice that had not been exposed to Tamoxifen and were plated in phenol-free Matrigel with one group receiving 4-hydroxy-tamoxifen (4OHT) throughout the 7-day-culture period. Previous experience had shown that 4OHT had no demonstrable effect on organoid formation for crypt nests isolated from WT mice (*n*=15; data not shown). MTT growth assay (Figure 5) showed that *creb*KO significantly impedes organoid growth. These effects were similar to those observed in Myb hypomorph (*Myb*^*Plt4/Plt4*^) organoid formation,^23^ suggesting that CREB loss does affect stem/progenitor cell proliferation when evaluated *ex vivo*.

When candidate cell cycle genes reported to be CREB targets were examined by qRT-PCR of total RNA isolated from parallel organoid cultures, no evidence of changes in *CyclinD1*, *CyclinA1*, *Erg1*^38^ or *Bcl-2*^39^ expression were observed (data not shown).

### CREB is elevated in intestinal adenomas and adenocarcinoma cells

**Figure 6:**
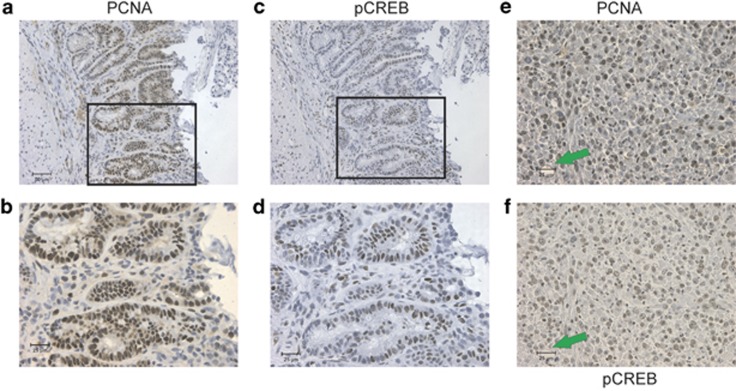
Proliferation and phospho-CREB in intestinal adenomas and colon adenocarcinoma are concordant. (**a** and **b**) *Apc*^*min/+*^ mice develop intestinal adenomas, which contain a large fraction of cells that are highly proliferative as shown by PCNA staining. (**c** and **d**) The pattern of PCNA staining mirrors that for phosphor-CREB. (**e** and **f**) MC38 colon adenocarcinoma cells form large tumors in syngeneic mice (C57Bl/6), which show high levels of PCNA+ve and pCREB+ve nuclei (green arrows indicate a focus to align nuclei in each micrograph)

As CREB loss leads to reduced proliferation in the GI and CREB has been shown to activate PCNA expression^36^ and impart oncogenic properties in some tissues,^35, 40^ we investigated CREB expression in tumor tissue. Intestinal adenomas from the *Apc*^*min/+*^ mouse were examined by IHC (Figures 6a and d) to show concordant PCNA and pCREB in the aberrant epithelial cells that align through consecutive sections. Furthermore, this relationship is also evident in mouse MC38 CRC cells (Figures 6e and f).

### Gene expression analysis using RNAseq

**Figure 7:**
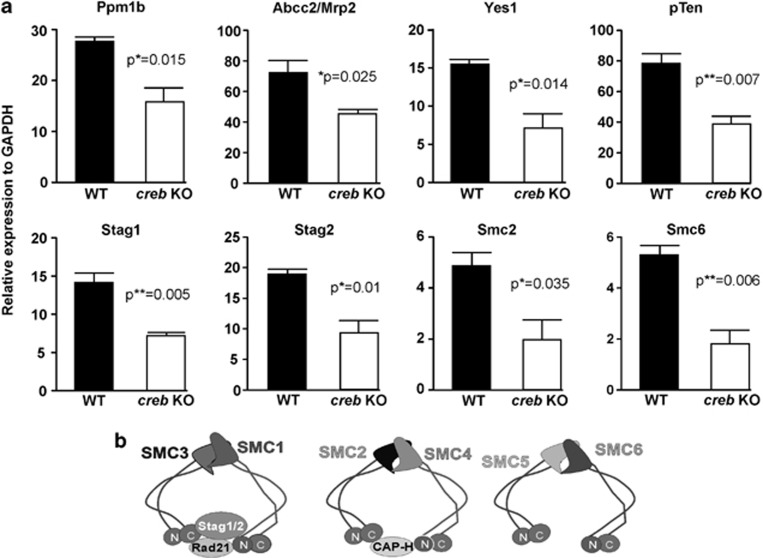
Messenger RNA expression changes in selected genes identified by RNAseq studies validated by qRT-PCR. (**a**) Significant changes in mRNA levels for phosphatases, *Pmm1b*, *pTen*, multidrug resistance gene *Abcc/Mrp2* and tyrosine kinase gene, *Yes1*. Four genes that encode proteins engaged in chromosome cohesion, Stag1, Stag 2, Smc2 and Smc6, are similarly lower in the *creb*KO small intestine crypts. Cartoons depicting the structural roles and general theme of interactions of the cohesion molecules are shown (**b**). Models of the structural convergence of several putative CREB target gene products, Stag1, Stag 2, Smc2 and Smc6, all of which are involved in cohesion functions, are shown

As the anticipated gene expression effects that might be expected in *creb*KO tissue were not observed, we isolated SI crypts for gene expression studies (these were depleted of the villi) from three *creb/villinCre*^*ERT2*^ *and* three *creb*^*fl/fl*^control mice where each cohort had been subjected to 4-week Tamoxifen treatment. This was important for both groups, as Tamoxifen influences transcription.^41, 42^ This strategy was also considered to most closely parallel that used to characterize the GI morphology, to examine the GI cells most likely to express pCREB and to capture unanticipated gene expression changes. This unbiased approach confirmed our previous data regarding no changes in the anticipated cell cycle genes (Supplementary Data File). However, several other genes showed significant changes that were confirmed by qRT-PCR, some of which had previously been suggested to be CREB target genes. Most notable was the lower expression of *pcna*, and of course *creb* itself. Among the genes showing the differential expression were phosphatases, Ppm1b and PTEN, tyrosine kinase Yes1 and cytotoxic chemotherapy drug transporter ABCC2/MRP2. We also noted that several chromatin cohesin genes stood out as being differentially reduced in the *creb*KO SI. These genes were thus evaluated by qRT-PCR as shown in Figure 7.

**Figure 8:**
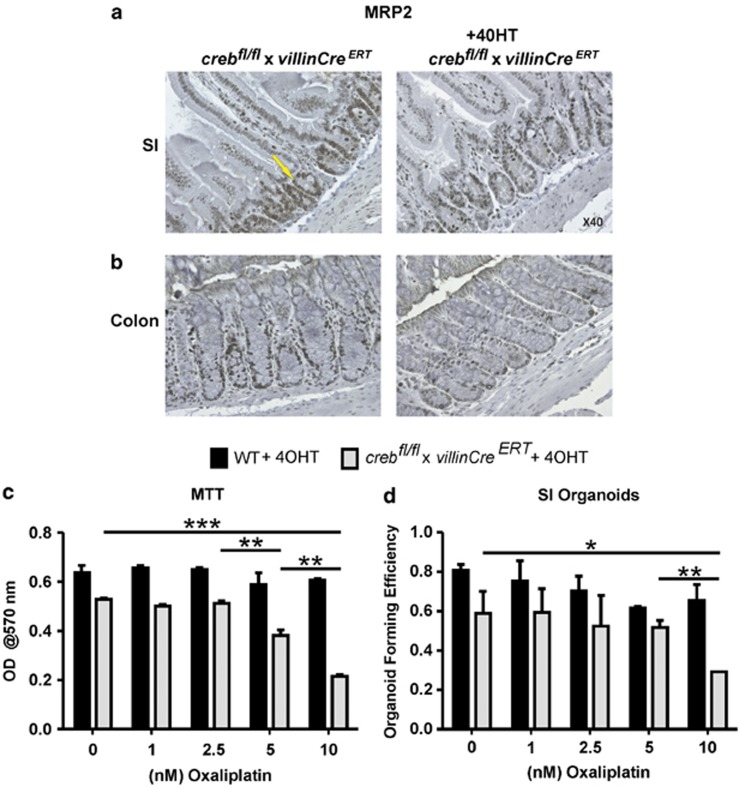
Multidrug transporter MRP2 is underexpressed in creb KO intestine. (**a**) Small intestine, and (**b**) colon sections were stained with *α*MRP2 antibodies (yellow arrow) to reveal reduced expression in *creb*^*fl/fl*^*/villinCre*^*ERT2*^crypts compared with WT, following 4-weeks exposure to Tamoxifen. (**c** and **d**) Organoid cultures were established in the presence of 4OHT for 3 days, and subsequently subjected to treatment with Oxaliplatin after cultures were allowed to proceed for another 7 days. These results show that as expected, the growth (MTT) and organoid-forming ability was impeded by *creb*KO alone, but these measures were significantly reduced compared with WT in the presence of Oxaliplatin. Means±S.E.M., two-tailed *t*-test. *P=0.01; ***P=0.001

To extend the analysis of the two genes most directly implicated in carcinogenesis that were expressed differentially in the *creb*KO SI, we employed antibodies to tumor suppressor protein, PTEN and drug transporter MRP2. Supplementary Figure 5 indicates that PTEN is expressed at a lower level in the *creb*KO, and most notably, in the cytoplasm and apical membranes in WT villi. MRP2 expression, however, was restricted to crypts in both the SI and the lower half of colonic crypts in WT mice and this signal was markedly diminished in KO mice (Figures 8a and b). Although MRP2 is reported to be apically expressed,^43, 44^ this was not overtly evident in WT sections. MRP2, unlike other MRPs, is elevated in CRC at the mRNA level^45^ and when adenomas and MC38 sections were examined for MRP2 protein, abundant expression was observed (Supplementary Figure 6). MRP2 is of substantive interest as frontline CRC cytotoxic drugs, Oxaliplatin, 5-Fluorouracil and Camptothecin are substrates or are influenced by MRP2 overexpression.^44, 45, 46^

To explore this relationship more directly, we established organoid cultures from WT and *creb*^*f/f*^*/villinCre*^*ERT2*^mice, allowing the culture to establish in the presence of 4OHT for 3 days prior to the addition of Oxaliplatin. As expected, the *creb*KO organoids grew less efficiently than the WT organoids, however, beyond this baseline difference, colony numbers and growth assessed by the MTT assay indicated that loss of CREB further sensitizes primary intestinal cells to the cytotoxic effects of the CRC chemotherapeutic drug (Figure 8).

## Discussion

A growing collective of TFs are involved in achieving intestinal crypt homeostasis and previously we have specifically shown a role of Myb,^20^ exploiting several Myb hypomorphic mutant mice,^18, 19^ sustained survival of which allowed the analysis of adult tissues not possible with embryonically lethal *Myb*KO mice^22^ and that complemented the inducible intestinal-specific *Myb*KO studies.^20^ Our discovery that the *p300*^*plt6/plt6*^ mutant in part phenocopied the *Myb* hypomorphs^17^ and indeed led to a substantial reduction in *Myb* expression highlights the utility of these mutants. That the *p300* mutation resides within the KIX domain raised a parallel question about the role of the archetypical p300/CBP-binding partner CREB in intestinal biology. Indeed, as transcription is mostly an orchestrated process of many TFs, we explored the interplay of p300 with Myb and CREB in regulating intestinal biology.

Protein (histone) acetyl transferases p300 and CBP are transcriptional co-activators and central to transcriptional activation of many genes through direct interaction with partner TFs that directly bind to enhancer elements within target gene promoter regions.^47^ Several interaction faces within p300/CBP are implied, with the KIX box being the best characterized.^48^ Of key relevance here is the kinase-inducible domain (KID) in CREB and the transactivation domain in Myb, which share structural similarities^49^ implying that Myb and CREB may even directly compete for KIX domains in p300/CBP. The intriguing observation that even though CBP expression is coincident with p300, it does not compensate for the defects associated with the p300 hypomorphic mutation, is also evident in the hematopoietic system.^50^

Evidence that p300 might be important in the intestines has been reported,^25^ with mRNA analysis in the rat indicating that p300 is expressed in excess of CBP.^24^ Here, we have shown by IHC that p300 is expressed to a similar extent to its close relative protein, CBP, within the crypt base and in the transit-amplifying regions. There is some debate about the relative contributions of each of these acetyl transferases, but the most convincing case is made for p300.^25^ Thus, the defects in the *p300*^*plt6/plt6*^ mutant leading to reduced proliferation, distorted differentiation and indeed shorter crypts are completely consistent with a central role for p300, and not for CBP.

When we examined the role of CREB in the intestines by tissue-specific deletion, it was anticipated that crypts would be either lost or replaced by crypts in which CREB was not deleted. This view was informed by our previous studies in high-proliferation tissues in mice and zebrafish,^8^ and other work that collectively shows that CREB is important in the maintenance of proliferation and protection from apoptosis. The *villin*Cre-driven deletion of CREB in both the colon and SI was essentially absolute and restricted to the epithelial cells, sparing cells in the *lamina propria* and *muscularis*. Nevertheless, near-normal looking crypts were evident, and directed investigation into proliferation and specific cell lineages revealed relatively subtle effects. For instance, by evaluating the PCNA+ve nuclei at each crypt cell position it became evident that proliferation could be maintained, but to a reduced extent. Such an effect was seen in the hypomorphic *p300*^*plt6/plt6*^ and *Myb* mutant mice,^20^ although these were not full loss of function mutants, whereas the *creb*KO was null. Similarly, an increase in mucin production and the presence of more ChrA+ve cells suggested that perhaps goblet and enteroendocrine cells may be abnormally increased, however, only goblet cells were increased and enteroendocrine cells were reduced in the hypomorphs.^20^ Together these data indicate that Myb has a more dominant role in crypt proliferation compared with CREB, and that p300 is required for Myb to prosecute this role, whereas CREB has mostly a distinct effect on the differentiation of secretory lineages.

The question remains as to why CREB loss has such a modest effect on this highly proliferative tissue? We were concerned that CREB loss might be compensated for by family members, CREM and ATF1, as reported in brain.^9^ However, these other family members were not expressed in the GI under homeostasis or when stressed, excluding compensation. Nevertheless, using RNAseq and by validation using qRT-PCR and IHC, a range of gene expression changes suggested that CREB regulates an important group of genes most relevant to carcinogenesis, for example, PTEN, MDR2 and, most interestingly, related proteins involved in chromatin/chromosome cohesion (for example, both STAG1 and 2 (SCC3; SA1 and 2) and SMC2, as well as SMC6). The established role of CREB in regulating these cohesion proteins in the context of DNA repair certainly warrants further investigation.

With regard to PTEN, it is fascinating that it is expressed at reduced levels in the *creb*KO crypts, as it is reported that the CREB protein is a target for PTEN phosphatase action,^51^ which opens up the prospect that a regulatory feedback loop may involve pCREB and PTEN.

Much has been made of the role of CREB in radiation sensitivity.^36^ Accordingly, we were surprised to find that deletion of CREB *in vivo* in a highly radiation-sensitive tissue like GI was innocuous. One clear difference between these contrasting observations might be that the radiosensitivity studies were done in cell lines with dominant negative forms of CREB^36^ and this may reflect that other mechanisms might be at play.

Reducing p300 function might be predicted to shift TF interactions to those with CBP as is argued for *β*-catenin,^52^ and this in turn would favor an expansion of non-differentiated (and perhaps) stem and progenitor cells. However, the opposite appears to be the case for the p300^*plt6/plt6*^ hypomorph, where reduced proliferation and enhanced differentiation occur. Overall, the data presented here suggest that much of the effects exerted by p300 in the GI are mediated through Myb and are also apparent in the hematopoietic system,^17, 19, 53, 54^ and, by contrast, CBP seems less important in both of these highly proliferative compartments.

The modest roles of CREB and, by implication, CREM and ATF1 in the GI was a genuine surprise, but there was evidence that pCREB/CREM/ATF1 is elevated in early-stage and advanced adenocarcinoma in the mouse. Commensurate with this association is the increased expression of a key cytotoxic drug transporter, MRP2, which may have a role in the response of CRC to frontline drugs, Oxaliplatin, 5-Flurouracil and Camptothecin. Based upon other studies where increased MRP2 may invoke resistance to cytotoxic drugs,^46^ it is reasonable to speculate that the elevated pCREB evident in mouse adenomas and CRC may also occur in human CRC and have a role in compromising the therapeutic impact of these drugs. Overall, the data presented here indicate that Myb is central to the action of p300 in the GI and that if CREB is working through p300, it is more engaged in potentially important gene regulation that impinges upon CRC but not GI homeostasis or radiation response.

## Materials and Methods

### Mice

*creb/villinCre*^*ERT2*^ mice were generated by crossing *creb*^*f/f*^ mice, as described,^9^ to *villinCre*^*ERT2*^mice^55^ on a C57BL/6J background. Mice were housed in a specific pathogen-free (SPF) facility and fed a standard animal diet in Peter MacCallum Cancer Centre. For induction of *creb* recombination, 5-week old *creb/villinCre*^*ERT2*^ mice were fed chow *ad libitum* supplemented with 0.1 g Tamoxifen citrate salt (Sigma, Australia, Castle Hill, NSW)/120 g food for 4 weeks. Littermates continued to be fed with a standard chow. The *p300*^*plt6/plt6*^ mutant mice were generated as described^17^ and maintained at the Walter and Eliza Hall Institute. *Apc*^*Min/+*^ mice^56^ were maintained on a C57BL/6J background. All animal experiments were approved by the Animal Ethics Committee of the Peter MacCallum Cancer Centre. The carcinogen-induced colon carcinoma cell line MC38 is described elsewhere, and was injected subcutaneously in C57Bl/6 mice to generate tumors.^57^

### Antibodies

Primary antibodies used in this study were: *α*CREB (1 : 500) (Cell Signaling, USA 9197), *α*pCREB (1 : 250) (Cell Signaling, Danvers, MA, USA, 9198), *α*Chromogranin A (ChrA), (1 : 100) (Santa Cruz Biotechnology, Santa Cruz, CA, USA H-300), *α*PCNA (1 : 100) (BD Biosciences, Bedford, MA, USA), *α*Neurogenin 3 (NGN3), (1 : 700) (Santa Cruz Biotechnology, M-80), *α*ATF2 (1:200) (Cell signaling), *α*CREM (1 : 100) (Abcam, Cambridge, MA, USA), *α*CBP, (1 : 100) (Santa Cruz Biotechnology, C-1), *α*Myb1.1 (Upstate Biotech, Lake Placid NY, USA), *α*p300 (1 : 800) (Santa Cruz Biotechnology, C-20), *α*MRP2 (1 : 500) (Santa Cruz Biotechnology, no. SC-5770), *α*pTen (1 : 300) (Cell Signaling, CS-9559). Secondary antibodies used were: anti-rabbit Impress reagent kit peroxidase (Vector labs, Burlingame, CA, USA), Envision system labeled polymer-HRP anti-mouse (Dako, Campbellfield, VIC, Australia), donkey-anti-goat IgG-HRP (Santa Cruz Biotechnology).

### Irradiation

*creb/villinCre*^*ERT2*^ mice treated with Tamoxifen for 2 weeks were exposed to a sublethal dose of *γ*-irradiation of 13 Gy. As irradiation compromises the immune system, 2.5 ml of 0.25% (w/v) Neomycin Sulfate and 0.13% (w/v) Polymyxin B Sulfate in dH_2_O were added to the drinking water of the mice in the days after irradiation. On day 5, the mice were culled, and the SI and colons were isolated and processed for histology.

### Histology and Immunohistochemistry (IHC)

SI and colons (*creb/villinCre*^*ERT2*^; *p300*^*plt6/plt6*^), and carcinomas from C57Bl/6J mice injected with the MC38 cell line were isolated and fixed in Methacarn (60% methanol, 30% chloroform, 10% acetic acid), dehydrated in a graded series of ethanol from 70 to 100% and then immersed in xylene. The tissues were embedded in paraffin, and 4-*μ*m sections were cut with the paraffin microtome and transferred onto 3-aminopropyl-triethoxy silane-coated glass slides.

For IHC, sections of SI and colon were dewaxed and dehydrated by immersing in xylene and a graded series of ethanol. Slides were boiled either in citrate buffer (10 mℳ tri-sodium citrate, pH 6.0) (CREB, pCREB, PCNA, ChrA), EDTA buffer (1 mℳ EDTA disodium salt dehydrate, pH 8.0) (NGN3, CREM) or TRIS buffer (10 mℳ TRIS, pH 9.0) (p300, CBP) for 3 min at 125 °C and 10 s at 90 °C in the Dako pressure cooker. Endogenous peroxidases were then blocked with 3% (v/v) H_2_O_2_ for 10 min. Slides were washed in Tris-buffered saline-Tween-20 (TBS-T) (5 M Tris, 0.15 M NaCl, pH 7.6, with 0.1% (v/v) Tween-20), and for p300, blocked in 5% (w/v) bovine serum albumin in TBS-T for 30 min. Then antibodies were added in TBS-T and incubated for 1 h at RT (PCNA, ChrA, CBP, p300) or O/N at 4 °C (CREB, pCREB, NGN3, CREM). Sections were washed with TBS-T and incubated with the corresponding secondary antibody for 30 min at RT. After washing in TBS-T, sections were exposed to 3,3′-diaminobenzidine tetrachloride (DAB+ substrate chromogen system) (Dako) for 1–10 min, resulting in a positive brown staining visible under the light microscope. For mucin staining, sections were exposed to periodic acid for 10 min followed by incubation with Schiff's reagent (PAS staining) (Australian Biostain, Traralgon, VIC, Australia). Slides were counterstained with hematoxylin for several seconds. After washing in water, slides were dehydrated in a graded series of ethanol, immersed in xylene and mounted with DPX.

### Organoid culture

SI of mice (*creb*^*lox/lox*^*;villinCRE*^*ERT2*^ and control littermates; between 3 and 5 weeks old) were isolated, opened longitudinally and cleaned in phosphate-buffered saline (PBS) supplemented with 0.1 mg/ml Nystatin (Sigma-Aldrich, St. Louis, MO, USA) and 0.1 mg/ml Gentamicin (Invitrogen, Carlsbad, CA, USA) (PBS-GN). The isolated intestines were incubated two times 15 min in PBS-GN on a shaker at 4 °C, followed by incubation in PBS containing 2 mℳ EDTA for 30 min at 4 °C. Then the tissues were shaken vigorously in 20 ml cold PBS, of which the supernatant was discarded. The tissues were transferred to 10 ml of cold PBS and shaken vigorously again to obtain fraction 2. This step was repeated to obtain fraction 3. Fractions 2 and 3 were combined and spun down at 200 × *g* for 3 min after which the supernatant, containing single cells, was removed. The pellet was resuspended in PBS and filtered through a 40-*μ*m filter. The required amount was spun down at 400 × *g* for 6 min. Five hundred or 1000 viable crypts were seeded in 50 *μ*l Matrigel Basement Membrane Matrix (BD Bioscience) per well of a 24-wells plate. The 24-wells plate was then placed at 37 °C for 30 min to allow polymerization. Control crypts were cultured in crypt culture medium consisting of Dulbecco's Modified Eagle Medium nutrient mixture F-12 (DMEM/F12; Sigma) supplemented with 10% (v/v) fetal calf serum (FCS), 100 U/ml penicillin and 100 U/ml streptomycin, 1 ng/ml recombinant mouse basic-FGF (eBioscience, San Diego, CA, USA), 2 ng/ml murine EGF (Peprotech, Rocky Hill, NJ, USA), 1 ng/ml recombinant mouse R-spondin-1 (R&D System Minneapolis, MN, USA), 0.1 ng/ml murine Noggin (Peprotech), B-27 Supplement Minus AO (Invitrogen). Half of the crypts of each mouse group were cultured in crypt culture medium supplemented with 0.1 *μ*g/ml 4OHT. Medium was changed every 2–3 days, and after 7 days, the cells were isolated by incubation with 50 *μ*l of Accumax (Millipore, Temecula, CA, USA) for 30 min at 37 °C.

Oxaliplatin (Ebewe Pharmaceutical Company, Touristic Area, Egypt) was dissolved in sterile dH_2_O and was added on day 3 following 4OHT (10^−7^ M) (Sigma). Organoids were processed as described previously.^23^

### RNA isolation, DNAse treatment and complementary DNA synthesis

For isolation of RNA from cells of cultured organoids RNeasy mini kit (QIAGEN, Hilden, Germany) was used according to the manufacturer's instructions. RNA was DNAse treated by adding 10 *μ*l 10 × DNAse1 Buffer (Promega, Madison, WI, USA), 3 *μ*l RQ1 RNase-Free DNase1 (1 U/*μ*l; Promega), 0.5 *μ*l RNAsin (40 U/*μ*l; Promega), 5 *μ*l DTT (0.1 M; Invitrogen) and 59 *μ*l H_2_O, prior to incubation at 37 °C for 30 min. Then, 100 *μ*l H_2_O was added followed by 200 *μ*l RNA-buffered phenol-chloroform (pH 5). The samples were spun down at 13 000 r.p.m. for 5 min, and an equal volume of 4% (v/v) isoamyl ethanol in chloroform was added to the top phase. After another spin at 13 000 r.p.m. for 5 min, the top phase was precipitated with three equal volumes of ethanol/sodium acetate (3% (v/v) 3 M NaAC; pH 5.6; in ethanol) O/N at −80 °C. Two microliter of glycogen (20 mg/ml) was added for visualization of the pellet. After incubation a spin at 13 000 r.p.m. for 15 min followed and the resulting pellet was washed in 70% (v/v) ethanol. Then the samples were spun down at 7800 r.p.m. for 8 min, the pellet was air dried for 5–10 min and dissolved in H_2_O. RNA concentrations were quantified with the nanodrop.

Complementary DNA (cDNA) was synthesized by incubating 2 *μ*g RNA with 0.5 *μ*l random primers (500 *μ*g/ml) (Promega) and 2 *μ*l 5 mℳ dNTP for 5 min at 65 °C. After incubation on ice for 1 min, a reaction mixture of 4 *μ*l 5 × First strand buffer (Invitrogen), 2 *μ*l 0.1 M DTT, 1 *μ*l RNAsin (40 U/*μ*l) and 1 *μ*l Superscript III Reverse Transcriptase (200 U/*μ*l; Invitrogen) was added and the samples were incubated for 10 min at RT, after which an incubation of 60 min at 50 °C followed. For inactivation of the reverse transcriptase reaction, the mixtures were incubated for 10 min at 70 °C.

### RNA sequencing

Large RNA was extracted from SI crypts using the miRNeasy mini kit and RNeasy MinElute Cleanup kit (QIAGEN). RNA concentration and quality was determined to be suitable using NanoDrop spectrophometer (Thermo Scientific, Wilmington, DE, USA) and integrity verified using the RNA 6000 kit (Agilent Technologies, Santa Clara, CA, USA). Library preparations were performed using the TruSeq RNA Sample Preparation protocol (Illumina, San Diego, CA, USA) and correct size established using the DNA 1000 kit (Agilent technologies). Libraries were quantified with qPCR, normalized and pooled to 2 nM before sequencing with single ended 50 bp reads using standard protocols on the HiSeq2000 (Illumina, San Diego, CA, USA). Computational resources were provided by Galaxy Project at Penn State.^58, 59, 60^

### qRT-PCR

A mixture of 0.8 *μ*l of cDNA, synthesized as described before, was combined with 10 *μ*l of SyBr Green PCR Master Mix (Applied Biosystems, Carlsbad, CA, USA), 2 *μ*l of 2 mℳ forward and reverse oligonucleotides (Geneworks, Adelaide, SA, Australia) and 7.2 *μ*l of H_2_O, and amplified in the StepOnePlus Real-Time PCR system (Applied Biosystems). Amplification conditions used were: 95 °C for 10 min, followed by 40 cycles of 95 °C for 15 s and 60 °C for 1 min, and a final cycle for the melt curve at 95 °C for 15 min, 60 °C for 1 min and 95 °C for 15 s. Expression levels of all genes were compared with GAPDH expression in order to determine relative mRNA levels. Primer sequences used for quantitative RT-PCR are tabulated in Supplementary Table 1

## Abbreviations

AB: alcian blue
CREB: cyclic-AMP response element-binding protein
PAS: periodic acid Schiff
PCNA: proliferating cell nuclear antigen
SI: small intestine
ISC: intestinal stem cell

## Supplementary Material

Supplementary Figure 6

Supplementary Excel spreadsheet
