# Supplementary figures and images for: The Myb-p300-CREB axis modulates intestine homeostasis, radiosensitivity and tumorigenesis
Source: Cell Death Dis. 2013 Apr 25;4(4):e605–. doi: 10.1038/cddis.2013.119 (PMC3641342; doi:10.1038/cddis.2013.119)

CBP

a

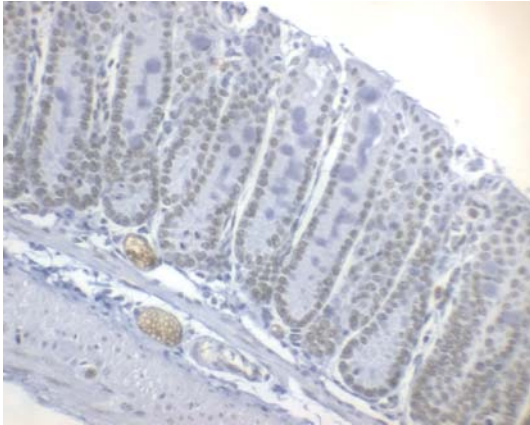

b

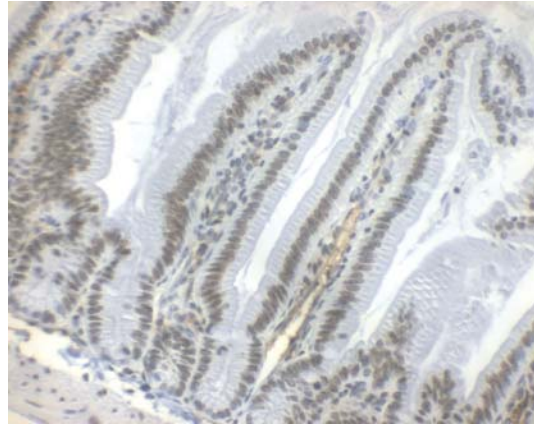

p300

c

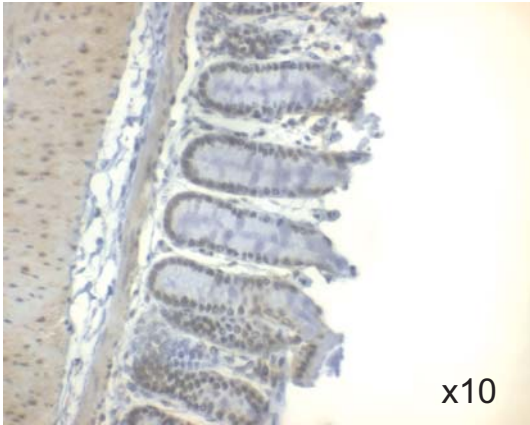

d

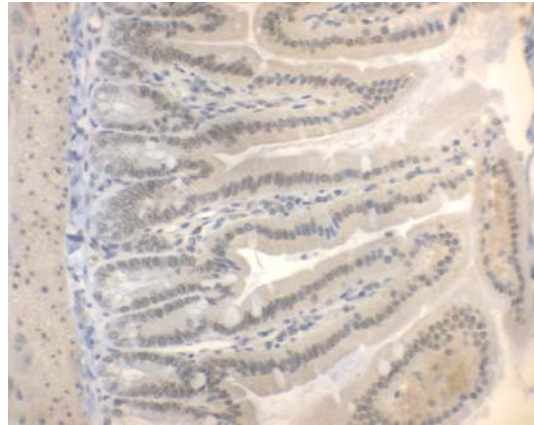

Supplement: Supplementary Figure 1 [file cddis2013119x1.pdf]

## ChrA/PAS

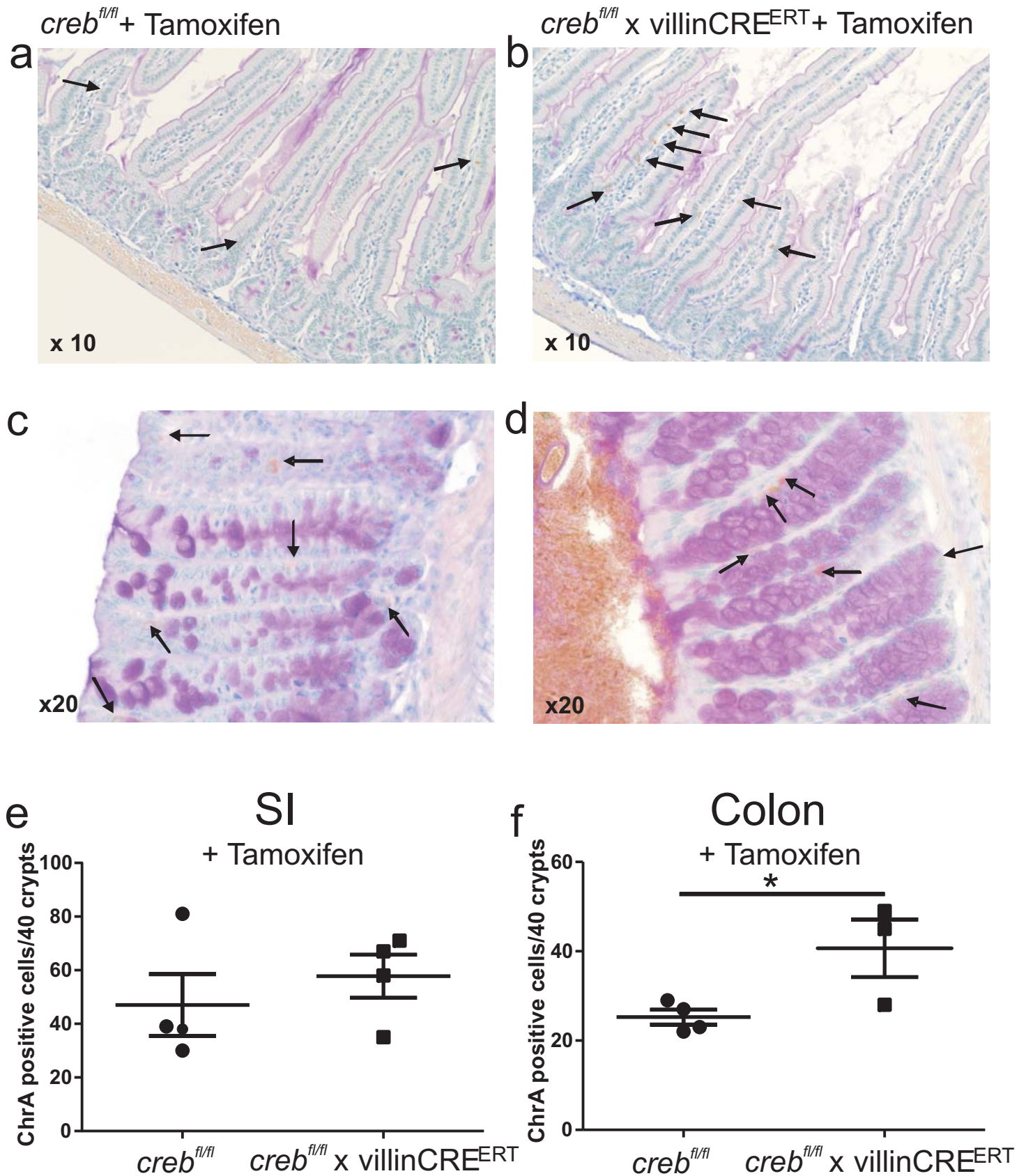

Supplement: Supplementary Figure 2 [file cddis2013119x2.pdf]

## ATF2

*creb<sup>fl/fl</sup>* + Tamoxifen

a

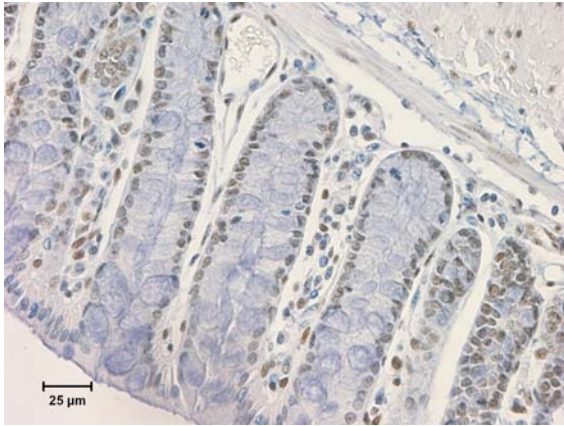

b

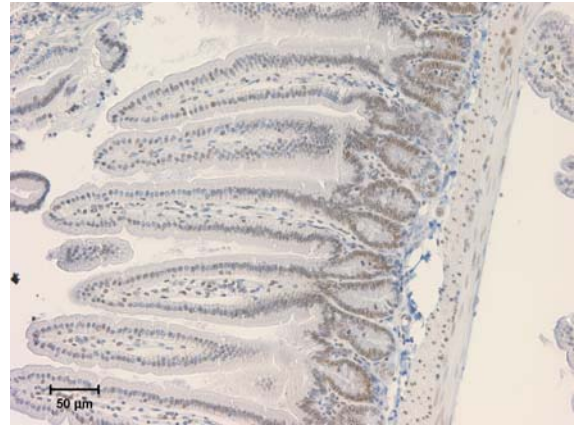

*creb<sup>fl/fl</sup>* x villinCRE<sup>EERT</sup> + Tamoxifen

d

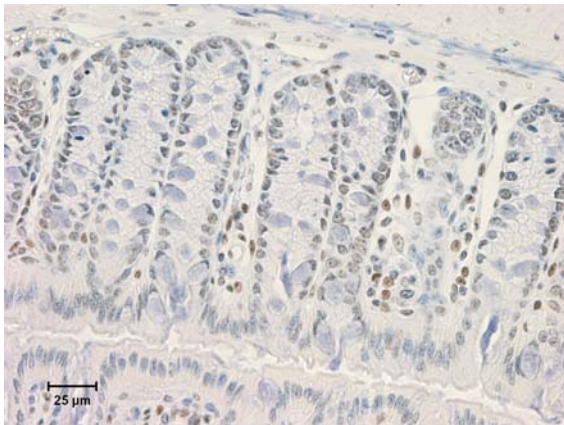

c

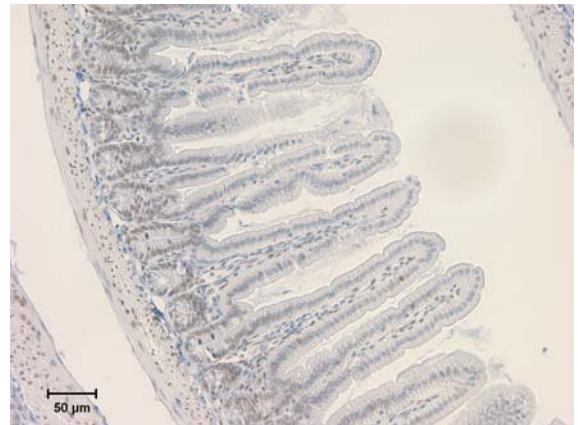

Supplement: Supplementary Figure 3 [file cddis2013119x3.pdf]

## Myb

a

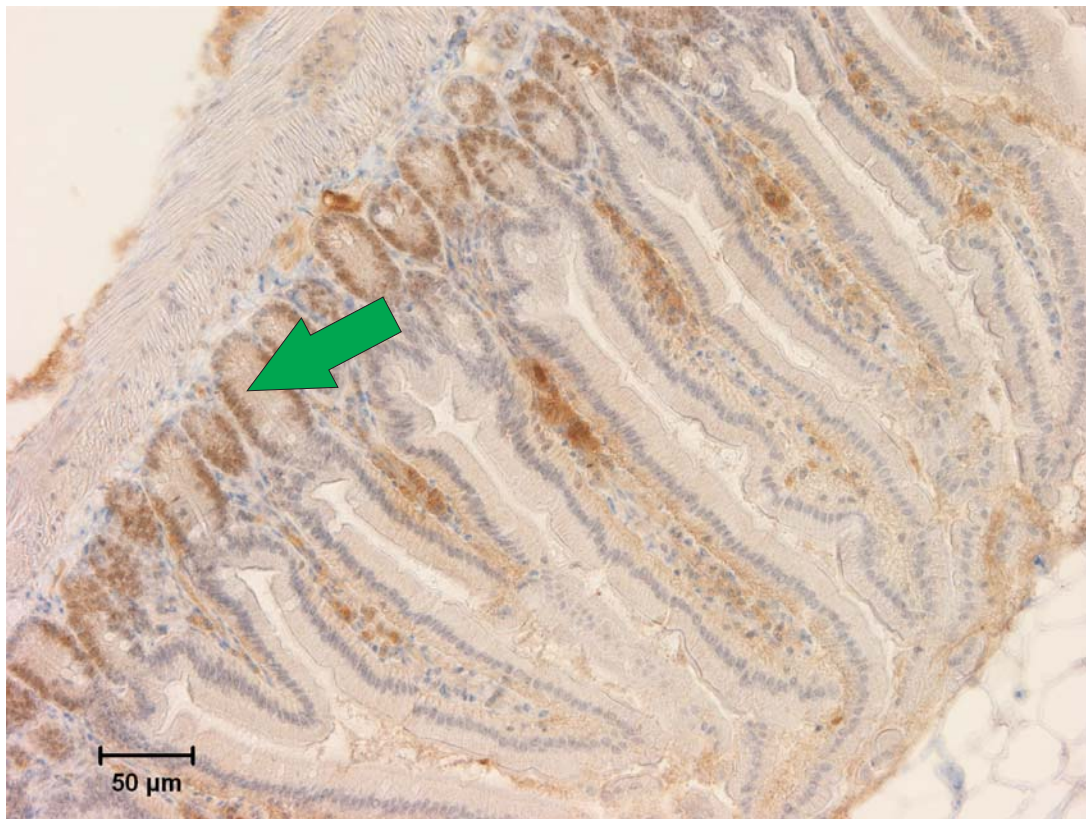

b

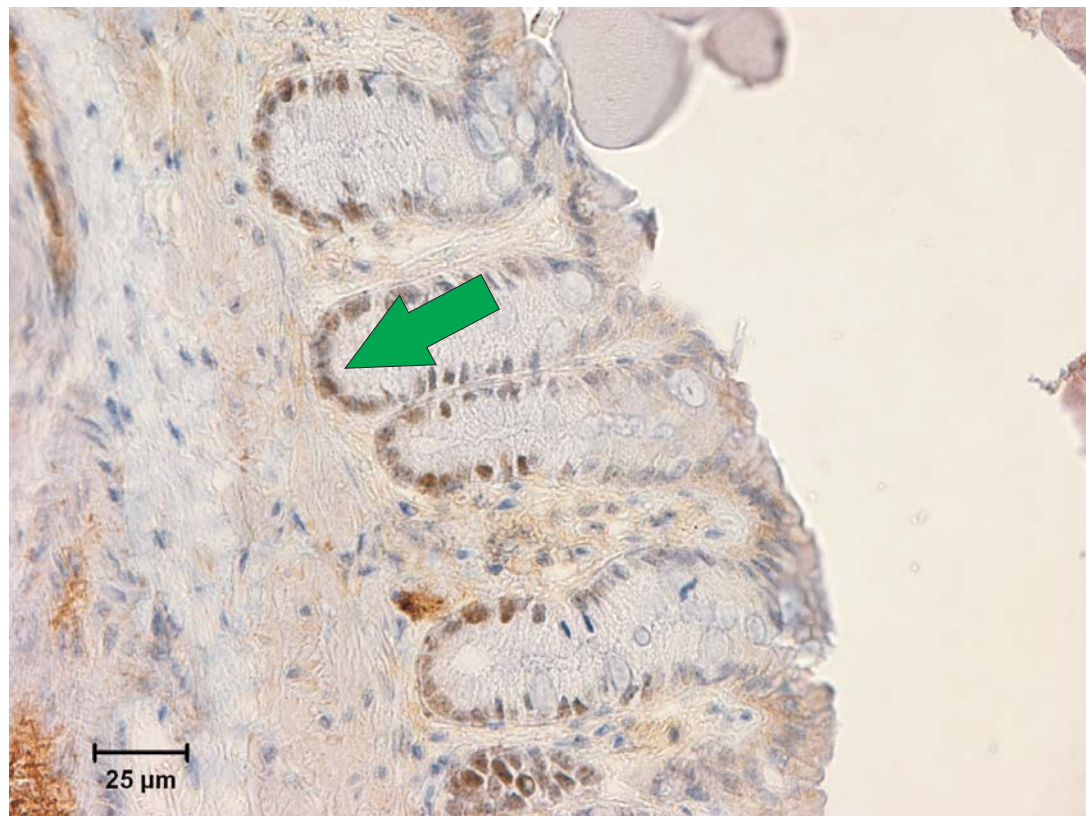

Supplement: Supplementary Figure 4 [file cddis2013119x4.pdf]

## MRP2

*APC*<sup>min/+</sup> adenoma

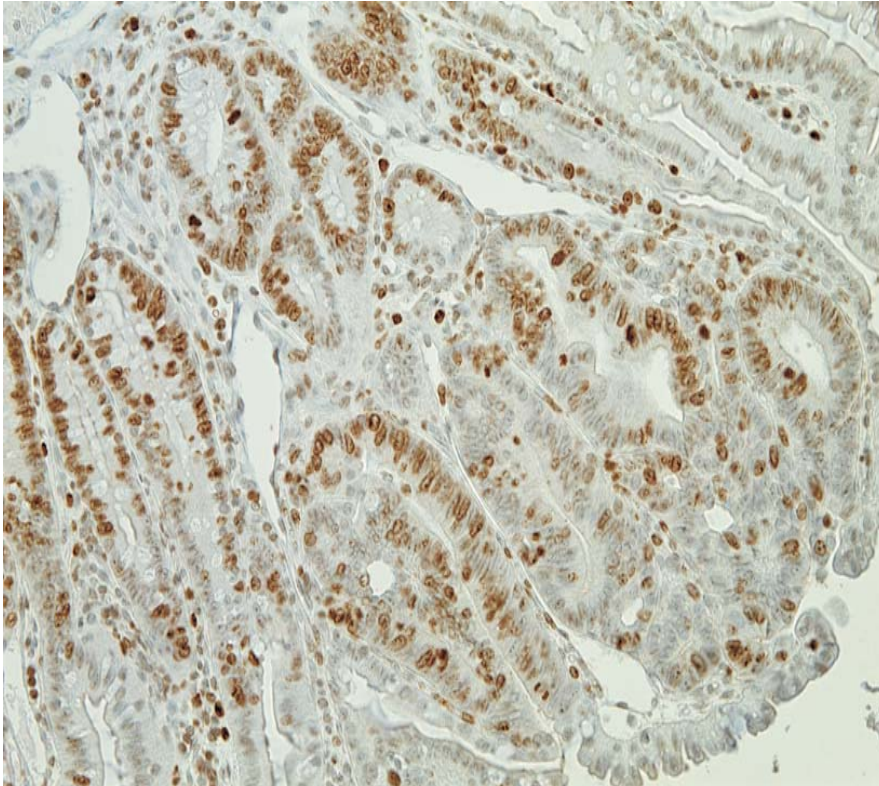

MC38 adenocarcinoma

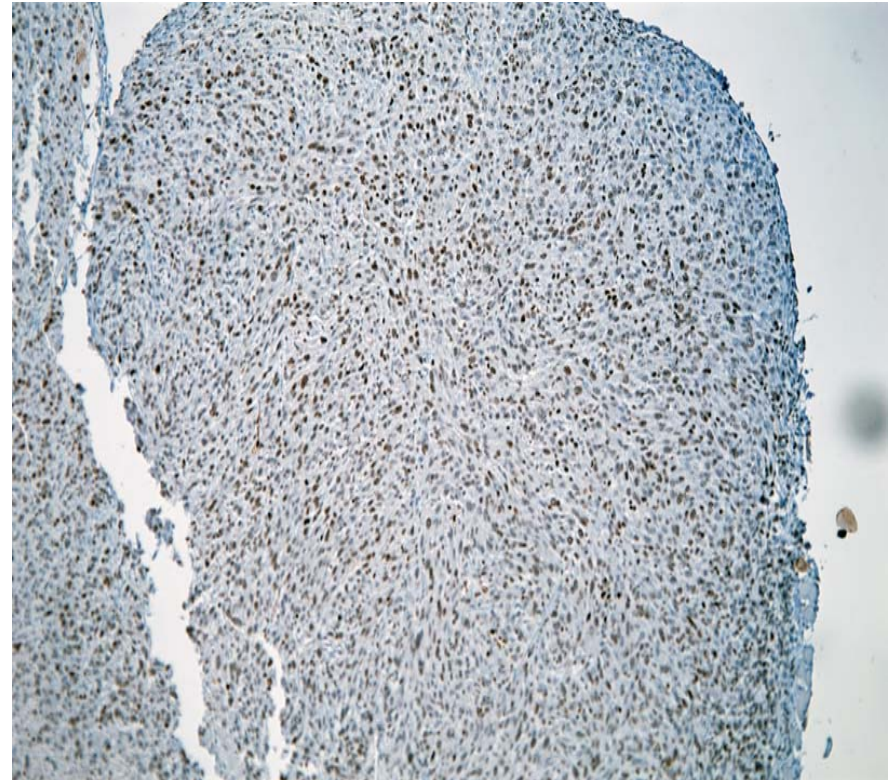

Supplement: Supplementary Figure 6 [file cddis2013119x6.pdf]
