# Supplementary material for: The Myb-p300-CREB axis modulates intestine homeostasis, radiosensitivity and tumorigenesis
Source: Cell Death Dis. 2013 Apr 25;4(4):e605–. doi: 10.1038/cddis.2013.119 (PMC3641342; doi:10.1038/cddis.2013.119)
Supplement: Supplementary Figure 5 [file cddis2013119x5.pdf]

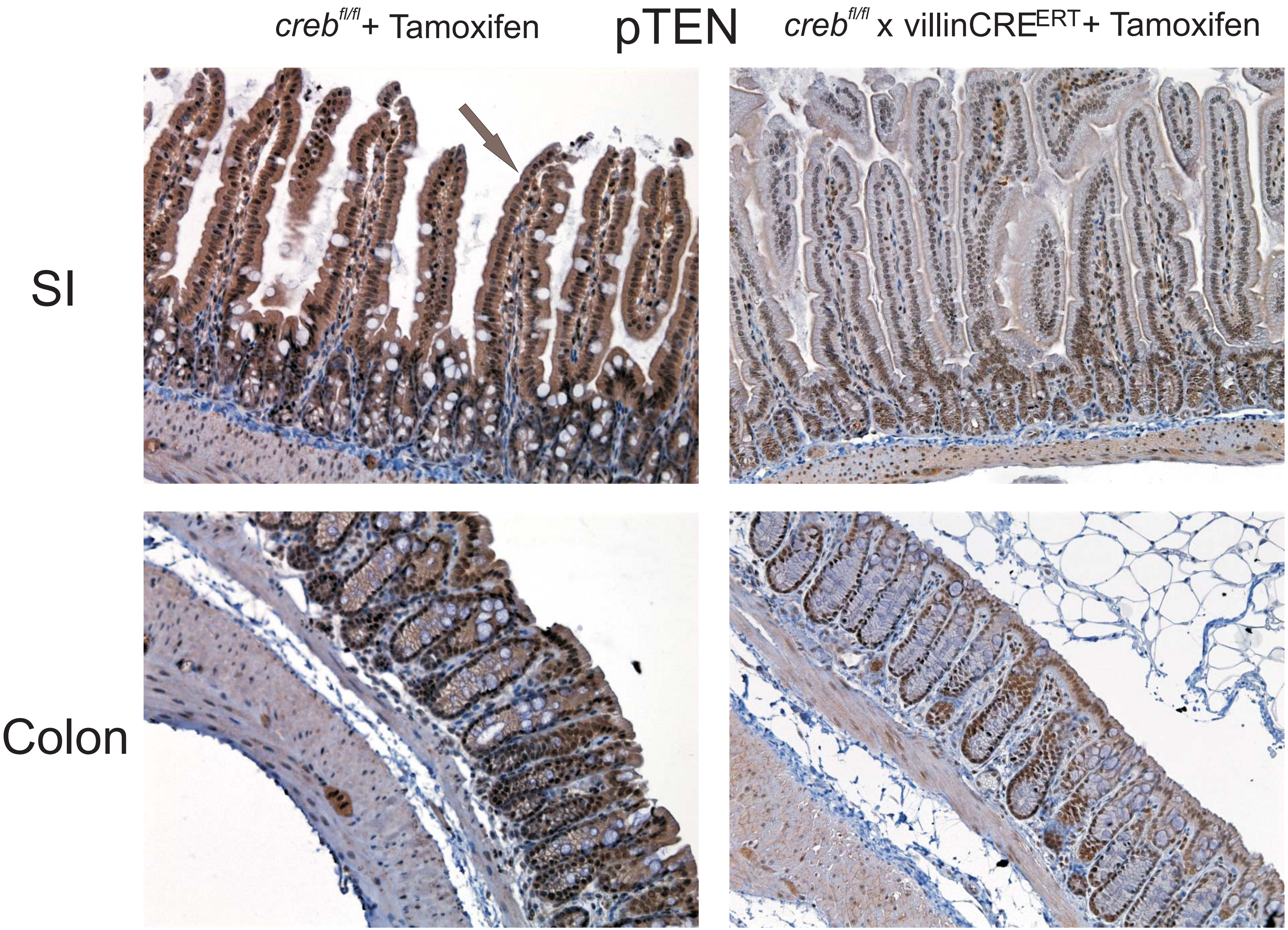

Supplementary Figure 5 Sampurno *et al*
