# Supplementary material for: The Myb-p300-CREB axis modulates intestine homeostasis, radiosensitivity and tumorigenesis
Source: Cell Death Dis. 2013 Apr 25;4(4):e605–. doi: 10.1038/cddis.2013.119 (PMC3641342; doi:10.1038/cddis.2013.119)
Supplement: Supplementary Table 1 [file cddis2013119x8.doc]

**Supplementary Table 1. Primer sequences used for quantitative RT-PCR**

| **Gene** | **Forward 5’** | **Reverse 5’** |
| --- | --- | --- |
| *gapdh* | GTATGACTCCACTCACGG | GGTCTGGCTCCTGGAAGA |
| *creb* | TGCCTCAGGCGATGT ACAAAC | GCACTGCCACTCTGTTCTCTA |
| *crem* | GCCAC AGGTGACATGCCAACTTAC | AGCAAATGTCTTTCAAAGTTTCAA |
| *Bcl-2* | CGCTAGGTGACCCCATTCTTC | AAGGCTTCT GGATTCCAGGAA |
| *cyclin A2* | TCAGGAAGACCAAGAGAATG | GGATAGTCAAGAGGTGTCAG |
| *Cyclin D1* | CTGGCCATGAACTACCTGGA | ATCCGCCTCTGGCATTTTGG |
| *cox-2* | AGAAGGAAATGGCTGCAGAA | CCCCAAAGATAGCATCTGGA- |
| *NGN3* | CTGCTCTTCTCTTAACTCTC | CAACACTGGA TTAGGTCAC- |
| *PCNA* | TGTGGAGCAACTTGGAATC | CTAAGGTCTCGGCATATACG |
| *Pten* | GACGGACTGGTGTAATGATTTGTG | CCTCTGACTGGGAATTGTGACTC |
| *MRP2* | CAGAGTCAGAGCCAAGATGTC | GAGAGCCTTCACCAACCAG |
